# Impact of psychological distress and psychophysical wellbeing on posttraumatic symptoms in parents of preterm infants after NICU discharge

**DOI:** 10.1186/s13052-022-01202-z

**Published:** 2022-01-24

**Authors:** Serena Salomè, Giuseppina Mansi, Carmine V. Lambiase, Marta Barone, Valeria Piro, Marcella Pesce, Giovanni Sarnelli, Francesco Raimondi, Letizia Capasso

**Affiliations:** 1 Division of Neonatology and Neonatal Intensive Care Unit, Department of Translational Medical Sciences, University of Naples Federico II, Via Sergio Pansini 5, 80131 Naples, Italy; 2 Division of Gastroenterology, Department of Clinical Medicine and Surgery, University of Naples Federico II, Via Sergio Pansini 5, 80131 Naples, Italy

**Keywords:** Parental stress, NICU, Preterm infants, Psychophysical wellbeing, PTSD, Depression

## Abstract

**Backgorund:**

Parents after Neonatal Intensive Care Unit (NICU) hospitalization of preterm infant may develop psychopathological symptoms.

The aim of the study was to determine how parental stress and psychophysical wellbeing affect posttraumatic symptoms (PTTS) in parents during the first year after NICU discharge. Moreover, this study aimed to explore any gender-specific difference in psychological distress among mothers and fathers.

**Methods:**

Prospective study design from September 2018 to September 2019. 20 pairs of parents of preterm infants admitted to a tertiary-level NICU were enrolled. Primary outcome was evaluation of PTTS in parents of preterm infants at one year after NICU discharge through Impact of Event Scale- Revised. Secondary outcomes were: impact of parental stress, psychophysical wellbeing, anxiety and depression respectively through Parental Stressor Scale: NICU, Short Form Health Survey-36(SF-36), Self-rating Anxiety Scale and Self-rating Depression Scale.

**Results:**

Mothers experienced higher rates of PTTS than fathers across the first year after NICU discharge (55% vs 20%). Maternal avoidance symptoms were associated with perception of their own infant look. Emotional aspects linked to maternal role predicted 36,8% of their hyperarousal symptoms. Maternal PTTS severity was predicted by their social functioning. Paternal mental health was associated both with maternal and paternal intrusive symptoms.. Maternal stress was associated with paternal avoidance symptoms. Paternal mental health predicted their hyperarousal symptoms (40%) and PTSD severity (52%).

**Conclusions:**

Parents who experienced NICU hospitalization of their own infant are at heightened risk to develop psychopathological symptoms. According to our initial hypothesis, investigating parental psychophysical wellbeing, through SF-36, originally provides a valuable support to detect parents at higher risk to develop posttraumatic outcomes across the first year after NICU discharge. In addition, paternal depression deserves to be taken into account since hospitalization as it could impact paternal PTSD development. Finally, these findings provide an initial evidence of gender-related patterns in PTSD development and psychological distress among mothers and fathers across the first year of their infant.

## Introduction

Parents of preterm infants have to face many stressful factors during NICU hospitalization of their baby [1, 2] such as fear, anxiety and depression [3, 4]. These symptoms are worse as compared to parents of full-term infants [5]. Furthermore, psychological distress can persist even after NICU discharge [6]. In addition, parents who lived NICU experience may show high rates of post-traumatic stress disorder (PTSD) shortly after their baby’s premature birth [7]. PTSD is a psychopathological condition developed after exposure to a traumatic or stressful event. According to the fifth edition of the Diagnostic and Statistical Manual of Mental Disorders (DSM-5), symptoms may include avoiding trauma-related cues (e.g., certain thoughts or places), recurrent experiencing (e.g., disturbing thoughts, feelings about the past), negative thoughts concerning oneself and the surrounding world, an overwhelming feeling of guilt or difficulties in experiencing positive emotions and high rates of arousal [8]. However, how psychophysical wellbeing and psychological distress (i.e. depressive and anxious symptoms and stress) relate to the development of posttraumatic stress symptom (PTTS) subtypes in parents of preterm infants after NICU discharge is not well known. In addition, there is a lack of how these factors may differently affect maternal PTTS development as compared to paternal one. The aim of this study was to determine how parental psychological distress and psychophysical wellbeing affect PTTS development in parents during the first year after NICU discharge. We evaluated the role of both maternal and paternal psychophysical wellbeing during their stay in NICU as potential protective factor of posttraumatic symptoms at the routinely 1-year- follow-up visit. In contrast, maternal and paternal psychological distress were investigated as potential risk factor of PTTS after discharge. In addition, we aimed to detect any gender-related difference in PTSD development among mothers and fathers.

## Methods

From September 2018 to September 2019, 32 couples of parents (32 mothers, 32 fathers) of infants admitted to a tertiary-level NICU were enrolled to participate. We asked them to fill out some questionnaires about stress, psychophysical wellbeing, anxiety and depression during hospitalization in NICU. Moreover, parents were invited to fill out the same questionnaires plus one about PTSD during the routinely follow-up visit one year after discharge. Parents gave their informed consent. The main reasons for refusal to participate were no interest of the other parent in taking part in the study, not better-defined personal problems or lack of time. All questionnaires were administrated and scored by trained psychologists.

### Measures

#### Parental stressor scale: neonatal intensive care unit (PSS:NICU)

The questionnaire is composed of 34 items and divided into 3 subscales [9]: Sights & Sounds, Infant Look and Behaviour and Parental Role. Parents had to rate each item on a 5-point Likert scale ranging from 1 (not at all stressful) to 5 (extremely stressful) according to their stress at time of administration. An overall stress score is computed from the three subscales and it ranges from 0 to 5. In Italy, the scale demonstrated high internal consistency ranging from .79 to .89 [10].

#### Short form health survey (SF-36)

It is a 36-item self-report survey of patient health [11]. It encompasses eight subscales: physical functioning (PF), role physical (RP), bodily pain (BP), general health (GH), vitality (VT), social functioning (SF), role emotional (RE), and mental health (MH). These subscales measure two distinct factors: Physical Component Summary (PCS) and Mental Component Summary (MCS). The cut-off value < 50 was used in order to categorize parents with poor general health [12].

#### *Self-rating anxiety scale (SAS)* and *self-rating depression scale (SDS)*

They are two 20-item self-report questionnaires [13, 14] that evaluate anxious and depressive symptoms. The items are related to the week before administering the questionnaires, with higher scores corresponding to higher levels of anxiety and depression. Scores < 50 indicate absence of psychopathological disturbances, whilst anxiety and depression based on the results of the questionnaires are labelled as mild (50–59), moderate (60–69) and severe (> 70).

#### Impact of event scale- revised (IES-R)

IES-R is a 22-item self-report measure for PTSD according to DSM-IV criteria [15]. It evaluates subjective distress caused by traumatic events. IES-R consist of three subscales encompassing three psychopathological domains: avoidance, intrusion and hyperarousal. Each item is rated by the participants on a scale from 0 to 4 (0 = “not at all”, 1 = “a little bit”, 2 = “moderately”, 3 = “quite a bit” and 4 = “extremely”) according to the previous seven days. We used mean scores for each subscale that range from 0 to 4 in order to compare PTSD psychopathological domains in parents. Finally, according to the latest guidelines, we considered a maximum overall score above 32 as an index of suspicion of PTSD clinical diagnosis [16].

### Data analysis

Data analysis was performed using SPSS v.21 programme (IBM). Firstly, paired sample Wilcoxon test was run in order to check for any differences about stress, PTTS, anxiety and depression rates in both mothers and fathers during NICU hospitalization and at the follow-up visit. Subsequently, Spearman and Pearson correlations were performed among variables during NICU hospitalization and posttraumatic symptoms at the follow-up visit. Then, we used stepwise multiple regression analysis to test whether NICU hospitalization variables were predictive of posttraumatic symptoms at the follow-up visit.

## Results

Among the 32 couples of parents initially enrolled, 20 of them (62%) accepted to participate to the two-time points of the research. Characteristics of the sample are reported in Table 1.

**Table 1:** Descriptive statistics for the final sample of infants and their parents

Subjects:	Infants ***N*** = 23 Mothers ***N*** = 20 Fathers = 20
**Variables**	**Mean (SD; range)**
Mean Birthweight	1375 g (SD = 458.57; 760–2500 g)
Normal Birthweight	1 (4.3%)
Low Birthweight	5 (21.7%)
Very Low Birthweight	13 (56.5%)
Extremely Low Birthweight	4 (17.3%)
Gestational Age	31 w (SD = 2.99; 25 to 36)
Late Preterm	4 (17.3%)
Early Preterm	14 (60.8%)
Extremely Low Gestational Age	5 (21.7%)
Type of birth	
Natural	5 (25%)
Caesarean section	15 (75%)
Mean days spent in NICU	59 (SD = 42.7; 17 to 199)
Days spent with invasive and not invasive respiratory support	9 (SD = 17.3; 0 to 59)
Type of ventilation	
None	8 (34.7%)
Not invasive	7 (30.4%)
Invasive and not invasive	8 (34.7%)
Sex	
M	14 (70%)
F	6 (30%)
Maternal age	34 (SD = 6.6; 27 to 49)
Primary school graduation	0
Middle school graduation	2 (10%)
High school diploma	13 (65%)
University degree	5 (25%)
Mean infant’s age at Time 1	19.8 days (SD = 7.5; 10 to 31)
Mean infant’s age at Time 2	7.55 months (SD = 2.39; 6 to 12)

**Fig. 1:**
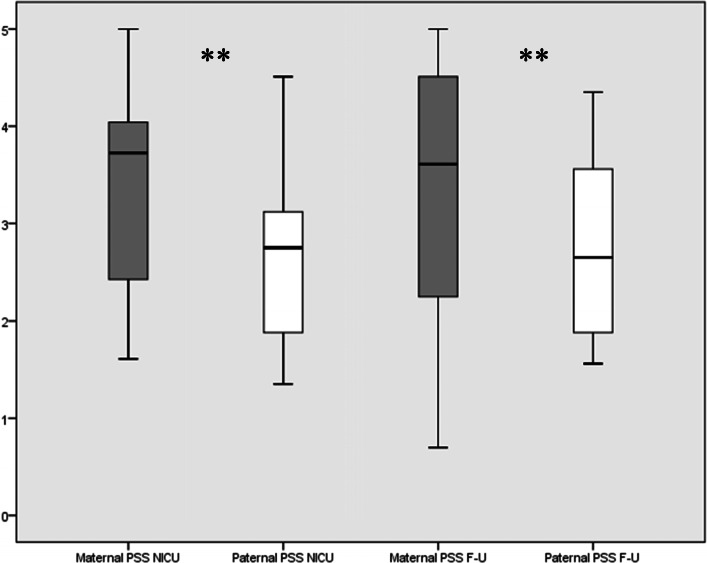
Comparison among maternal and paternal PSS:NICU scores comparison during hospitalization and at the follow-up visit. Wilcoxon paired test. **p < .01

**Fig. 2:**
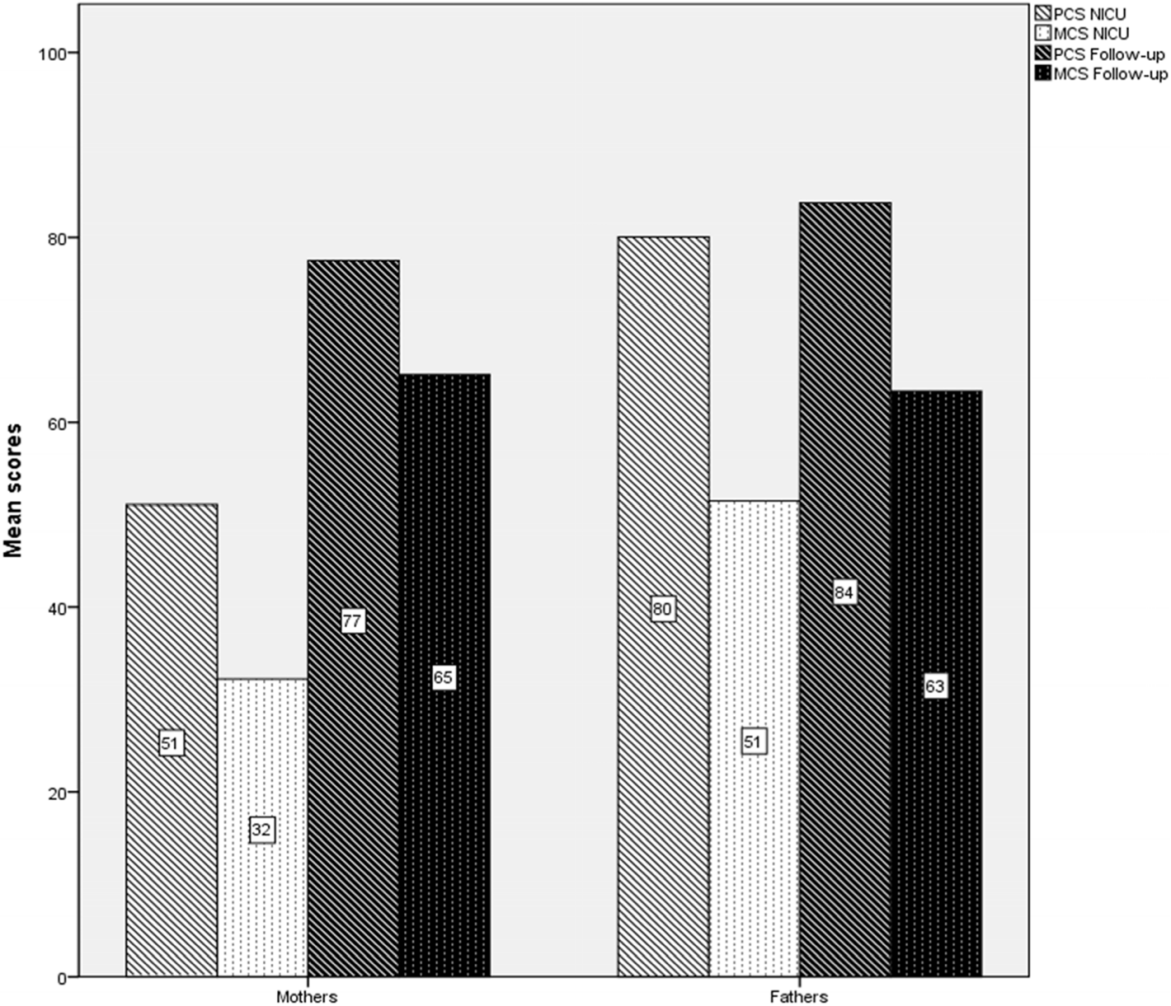
Physical and Mental health in parents during NICU stay and at the follow-up visit. Wilcoxon paired test

**Fig. 3:**
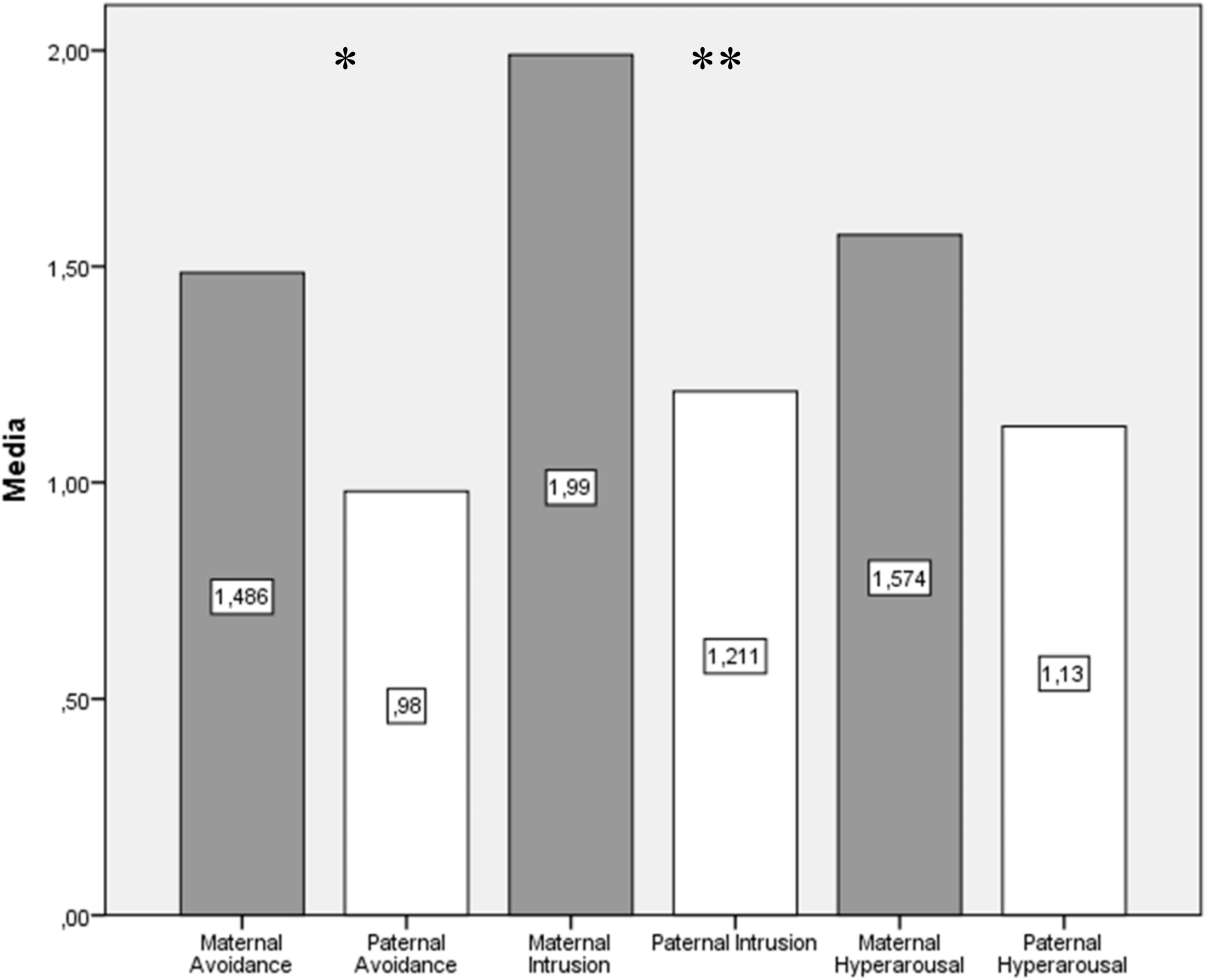
Posttraumatic types of symptoms in mothers as compared to fathers. Wilcoxon paired test. *p < .05. **p < .01

Maternal stress scores were higher than paternal ones both during NICU hospitalization (Z = -2.913, *p* < .01) and at the follow-up visit (Z = -2.875, *p <* .01; Fig. 1). Fathers showed better scores than mothers in both mental (Z = -2.466, *p* < .05) and physical health (Z = -2.192; *p <* .01) during NICU stay. Nevertheless, there was no significant difference among parents in assessments carried out several months after discharge (Fig. 2). Ninety percent of mothers and 65% of fathers screened positive for depression during NICU hospitalization. In particular, mothers reached higher scores than fathers (Z = -2.295, *p* < .05). Meanwhile, 80% of mothers and 60% of fathers screened positive for depression at the follow-up visit. Maternal (mean = 53, range = 34–69) and paternal (mean = 48, range = 25–65) depression scores were not significantly different at the follow-up visit (Z = -1.3702, *p* > .05). Mothers (65%) screened positive for anxiety more frequently as compared to fathers (45%). In addition, maternal scores were higher than their partner scores (Z = -2.466, *p* < .05). At the follow-up visit, 35% of mothers and 15% of fathers screened positive for anxiety disturbances. In particular, maternal anxiety score (mean = 48, range = 49–68) was significantly higher (Z = -2.668, *p* < .01) than paternal anxiety score (mean = 41, range = 25–63). Finally, 55% of mothers screened positive for PTSD diagnosis (i.e., IES-R score ≥ 33) as compared to 20% of fathers at the follow-up visit. In particular, mothers significantly scored higher than fathers in avoidance (Z = -2.513, *p* < .05) and intrusion subscales (Z = -2.854, *p* < .01; Fig. 3).

Correlation analysis among NICU questionnaires and maternal IES-R subscales are shown in Table 2.

**Table 2:** Spearman correlations among NICU variables and maternal IES-R subscales

***Follow-up***	Maternal Avoidance	Maternal Intrusion	Maternal Hyperarousal	Maternal Total score
***NICU***				
Sight & Sounds *m*	.449*	.480*	.380	.453*
Inf. Look & Behaviour *m*	.681**	.543*	.464*	.674**
Parental role *m*	.585**	.418	.306	.497*
PSS: NICU total score *m*	.584**	.532*	.337	.571**
Social activity *m*	−.577**	−.600**	−.532*	−.658**
Role Emotional *m*	−.442	−.564**	−.619**	−.544*
MCS *m*	−.436	−.516*	−.592**	−.566**
Social activity *f*	−.506*	−.453*	−.342	−.439
Mental Health *f*	−.421	−.616**	−.283	−.408
Vitality *f*	−.403	−.507*	−.312	−.354
MCS *f*	−.359	−.469*	−.191	−.273
SAS *m*	.486*	.527*	.314	.382

*m* = mother referred; *f* = father referred; *p < .05; **p < .01

Regression analysis showed that maternal IES-R Avoidance subscale was negatively predicted by maternal Social Functioning subscale (*R*^*2*^ = .328, β = −.572, t = − 2.962, *p* < .01) while maternal Infant Look & Behaviour subscale predicted 34,7% of variance of their IES-R Avoidance subscale (*R*^*2*^ = .347, β = .589, t = 3.090, *p* < .01). Maternal IES-R Intrusion subscale was predicted by their PSS:NICU total score (*R*^*2*^ = .316, β = .562, t = 2.885, *p* < .05) and anxiety as measured by SAS (*R*^*2*^ = .244, β = .494, t = 2.411, *p* < .05). Otherwise, paternal Mental Health subscale was the most predictive variable of maternal IES-R Intrusion subscale. In particular, lower scores at paternal Mental Health subscale were predictive of higher rates of maternal IES-R Intrusion subscale (*R*^*2*^ = .344, β = −.586, t = − 3.069, *p* < .01). Maternal Role Emotional subscale predicted 36,8% of their IES-R Hyperarousal subscale variance (β = −.607, t = − 3.241, *p <* .01). Maternal IES-R total score was predicted by their PSS:NICU total score (*R*^*2*^ = .374, β = .612, t = 3.283, *p* < .01). Nevertheless, maternal Social Functioning subscale was the most predictive variable explaining 39,5% of their IES-R total score (*R*^*2*^ = .395, β = −.628, t = − 3.425, *p* < .01).

Correlation analysis among NICU questionnaires and paternal IES-R subscales are shown in Table 3.

**Table 3:** Spearman correlations among NICU variables and paternal IES-R subscales

` ***Follow-up***	Paternal Avoidance	Paternal Intrusion	Paternal Hyperarousal	Paternal total score
***NICU***				
Sights & Sounds *m*	.564**	.471*	.424	.517*
Inf. Look & Behav. *m*	.671**	.600**	.417	.614**
Parental role *m*	.638**	.476*	.384	.565**
PSS: NICU *m*	.756**	.625**	.574**	.745**
Sights & Sounds *f*	.511*	.521*	.270	.529*
PSS: NICU *f*	.494*	.486*	.192	.495*
Vitality *f*	−.555*	−.661**	−.545*	−.654**
MCS *f*	−.584**	−.724**	−.579**	−.733**
SAS *m*	.449*	.322	.267	.413
SDS *f*	.705**	.676**	.572**	.766*

*m* = mother referred; *f* = father referred; *p < .05; **p < .01

With regard to regression analysis, paternal SDS positively predicted IES-R Avoidance subscale (*R*^*2*^ = .382, β = .618, t = 3.338, *p* < .01). Paternal Mental Component Summary was associated with their IES-R Avoidance subscale (*R*^*2*^ = .290, β = −.539, t = − 2.713, *p* < .05). Maternal PSS:NICU total score predicted 51,6% of paternal IES-R Avoidance subscale variance (*R*^*2*^ = .516, β = .718, t = 4.381, *p* < .01). Paternal SDS was positively associated with their IES-R Intrusion subscale (*R*^*2*^ = .437, β = 661, t = 3.739, *p* < .01). Further, maternal PSS:NICU total score was associated with paternal IES-R Intrusion subscale (*R*^*2*^ = .442, β = .665, t = 3.775, *p* < .01). Paternal Mental Component Summary predicted 49,5% of IES-R Intrusion subscale in fathers (*R*^*2*^ = .495, β = −.703, t = − 4.199, *p* < .01). Maternal PSS:NICU was predictive of paternal IES-R Hyper-arousal subscale (*R*^*2*^ = .266, β = .515, t = 2.552, *p* < .05). Moreover, paternal Mental Component Summary was significantly associated with their IES-R Hyper-arousal subscale (*R*^*2*^ = .400, β = −.421, t = − 2.839, *p* < .01)*.* Paternal IES-R total score was predicted by maternal PSS:NICU total score (*R*^*2*^ = .518, β =720, t = 4.398, *p* < .001) and paternal SDS (*R*^*2*^ = .492, β =701, t = 4.125, *p* < .01).

## Discussion

To our knowledge, this is the first study to investigate psychophysical wellbeing in both mothers and fathers of premature infants during baby’s first year of life by mean of SF-36 questionnaire. In addition, we examined the role of parental stress, psychophysical wellbeing, anxiety and depression during NICU hospitalization and their consequences on the development of PTSD types of symptoms in both mothers and fathers at the routinely one-year-follow-up visit.

Our findings underline that mothers experienced more mental and physical health challenges than fathers during NICU stay. This could be due to the fact that mothers of preterm infants usually have difficult deliveries and are rapidly separated from their offspring due to health-related adversities of premature birth while fathers can keep close to the new-born since the beginning. Conversely, maternal psychophysical wellbeing ameliorated at one-year-follow-up visit showing that NICU setting particularly influences psychophysical wellbeing in mothers.

Moreover, we found almost all mothers and a quite high percentage of fathers who screened positive for depression during NICU hospitalization. Importantly, this prevalence only slightly decreased at the follow-up visit. Thus, our findings underline the potential chronic role of depressive symptoms both in mothers and fathers. On the contrary, anxious symptoms even if characterized parental experience during NICU hospitalization, widely decreased during the first year after discharge in both mothers and fathers. However, mothers showed higher rates of anxious symptoms as compared to fathers at the follow-up visit. This result underlines that anxiety could be a chronic feature in mothers who experienced NICU hospitalization of their baby.

Based on our data, PTSD clinical diagnosis at the follow-up visit was more common in mothers than fathers (55% vs. 20%). This difference is in line with a previous study [17] that recruited a similar sample as well as used the same questionnaire to evaluate PTSS (i.e., 60% of mothers vs. 47% of fathers). However, our sample, particularly fathers, is less affected by PTTS after NICU discharge. This could be due to the fact that parents of our study were supported by the psychologist of NICU during their infant hospitalization in contrast with the latter study.

Kim et al. [18] argued as most of their NICU mothers never showed any PTSD symptom during the first year after discharge. However, they recruited mothers of high-risk infants including several medical conditions that require NICU hospitalization (e.g., preeclampsia, premature birth, infections). Due to the heterogeneity of this population, the prevalence of PTTS may differ compared to a homogenous group such as parents of preterm infants as described in our research.

As regard to clinical manifestations of PTSD, mothers were more sensitive to develop avoidant and intrusive symptoms as compared to fathers. This result extended the knowledge concerning how NICU hospitalization affects maternal experience with more traumatic outcomes as compared to fathers [19]. Interestingly, hyper-arousal symptoms did not differ among mothers and fathers in our study. This result originally suggests how reactions of alertness (e.g., sleeping problems, irritability, difficulties concentrating) could be a common signature in parents who faced the premature birth and NICU hospitalization of their infant. Further studies should investigate arousal regulation states among parents of preterm infants compared to parents of full-term ones. Furthermore, mothers used to experience negative emotions concerning lack of fitting to their parental role. This predicted disruption of their arousal regulation states after NICU discharge. Moreover, maternal negative perception of their infant look increased maternal avoidance symptoms and the risk of PTSD diagnosis after discharge. Our findings confirm that maternal post traumatic symptoms may be exacerbated by a negative perception of both the infant general condition and their role as caregivers [20]. In addition, our study underlines that mothers who experience lack of social support during NICU stay are at heightened risk to develop avoidance symptoms after discharge. Moreover, we found that mothers could have heightened risk for posttraumatic outcomes if their partner perceived lack of social support during NICU stay. Thus, screening for parents without social support of family and friends during hospitalization is crucial to identify parents at higher risk of posttraumatic sequelae. All these results underline the importance to boost participation of both mothers and fathers in joining NICU staff to take care of their premature infant as much as possible. Importantly, promoting parent-infant bonding since NICU stay could increase both parental health and skills to take care of their infant after discharge.

Additionally, we found gender-related differences among parents. In particular, anxiety in mothers and depression in fathers respectively increased maternal and paternal intrusive symptoms after NICU discharge. With regard to fathers, their depressive symptoms showed to be one of the core features of PTSD development several months after discharge. Noteworthy, their mental health issues and maternal stress increased paternal avoidant symptoms at the follow-up visit. This result is in line with an emerging evidence concerning paternal perinatal depression as a public health concern to take care of [21]. In particular, a number of studies is suggesting to develop specific tools in order to detect perinatal depression symptoms in fathers [22]. A systematic review of qualitative studies shows that paternal depressive symptoms during NICU admission may be explained by their oscillating self-representation and the fear to harm their own infant [23]. These feelings may affect paternal caregiving engagement leading to ambivalent and depressive mood. Thus, sustaining and reassuring fathers about both their spouse and infant health status and promoting their parental role during NICU stay is fundamental. Importantly our findings extend these studies adding the key role of paternal depressive symptoms in fathers of preterm infants since NICU hospitalization and their crucial role in PTTS development in both mothers and fathers during the first year after discharge.

As paternal depressive symptoms seem to follow different paths as compared to mothers, training NICU personnel for perinatal mental health issues of fathers is recommended.

Our findings confirm that gender-related differences are particularly relevant as they provide a complex framework explaining both maternal and paternal dynamic adjustment to preterm birth experience [24]. Importantly, both of them can disrupt family wellbeing and new-born care. Thus, our findings can lead to develop tailored interventions in order to fill parents needs since the first days in NICU through a family-centred care approach. In particular, we recommend to take care of paternal depressive symptoms and maternal perception of their own infant and parental role alterations as both these issues could disrupt parent-infant interactions and child development [25].

Finally, keeping parents in touch with their family members and friends could promote their perception of social support.

### Strengths and limitations

The strong points of our investigation include the longitudinal evaluation of numerous psychological and psychophysical domains by standardized tools in both mothers and fathers of preterm infants. We also shed a new light on the key role of parental psychophysical well-being and its consequences on posttraumatic outcomes.

Among the limitations of our study, we acknowledge the relatively small sample size, a large parental drop-out rate and the lack of parents of full-term infants as a control group.

## Conclusion

Parents who experienced NICU hospitalization of their own infant are at heightened risk to develop psychopathological symptoms. According to our initial hypothesis, investigating parental psychophysical wellbeing, through SF-36, originally provides a valuable support to detect parents at higher risk to develop posttraumatic outcomes across the first year after NICU discharge. In addition, paternal depression deserves to be taken into account since hospitalization as it could impact paternal PTSD development. Finally, these findings provide an initial evidence of gender-related patterns in PTSD development and psychological distress among mothers and fathers across the first year after NICU discharge. These findings underline the importance to promote parental psychophysical wellbeing since NICU stay, especially social support and paternal mental health as it could have an inestimable impact in order to prevent psychopathological outcomes due to NICU experience in both mothers and fathers.

## Data Availability

The datasets used and analysed during the current study are available from the corresponding author on reasonable request.

## Abbreviations

NICU: Neonatal intensive care unit
PTTS: Posttraumatic symptoms
PTSD: post-traumatic stress disorder
DSM-5: Diagnostic and Statistical Manual of Mental Disorders fifth edition
PSS: NICU: Parental stress scale: neonatal intensive care unit
PF: Physical functioning
RP: Role Physical
BP: Bodily Pain
GH: General Health
VT: Vitality
SF: Social Functioning
RE: Role Emotional
MH: Mental Health
PCS: Physical Component Summary
MCS: Mental Component Summary
SF-36: Short-Form Health Survey
IES-R: Impact of Event Scale-Revised
SAS: Self-rate anxiety scale
SDS: Self-rate depression scale
